# Challenges, Barriers, and Facilitators in Telemedicine Implementation in India: A Scoping Review

**DOI:** 10.7759/cureus.67388

**Published:** 2024-08-21

**Authors:** Simran Arora, Ramesh K Huda, Sakshi Verma, Mukti Khetan, Ramesh K Sangwan

**Affiliations:** 1 Public Health, Indian Institute of Public Health Gandhinagar, Gandhinagar, IND; 2 Information Technology, National Institute for Implementation Research on Non-Communicable Diseases, Indian Council of Medical Research, Jodhpur, IND; 3 Biostatistics, National Institute for Implementation Research on Non-Communicable Diseases, Indian Council of Medical Research, Jodhpur, IND; 4 Epidemiology and Public Health, National Institute for Implementation Research on Non-Communicable Diseases, Indian Council of Medical Research, Jodhpur, IND

**Keywords:** facilitators, challenges & barriers, implementation, esanjeevani, telemedicine

## Abstract

Telemedicine has revolutionized healthcare access by breaking geographical barriers and facilitating remote consultations. The eSanjeevani platform has been pivotal in India, especially during the COVID-19 pandemic. Despite its benefits, implementing telemedicine faces various challenges and barriers. This scoping review aims to identify these challenges, barriers, and facilitators in the Indian context.

This review follows the preferred reporting items for systematic reviews and meta-analyses extension for scoping reviews (PRISMA-ScR) guidelines. A comprehensive literature search was conducted using PubMed and Google Scholar to identify studies published between January 2019 and January 2024. Studies on telemedicine adoption, barriers, and facilitators in India were included. Data were extracted and synthesized from 26 quantitative, qualitative, and mixed-methods research studies.

Individual-level challenges include limited digital literacy, particularly among older adults and those in rural areas, and health literacy issues. Barriers such as limited smartphone access, unreliable internet connectivity, and socio-cultural preferences for in-person consultations were identified. Facilitators at this level include family involvement, training programs, and community outreach. Provider-level challenges involve a lack of training and concerns about care quality, while barriers include insufficient infrastructure and medico-legal concerns. Facilitators include ongoing training, clear guidelines, and user-friendly telehealth systems. System-level challenges include integrating telemedicine into existing infrastructure and ensuring data security. Barriers include inadequate funding and resistance to change, while facilitators include policy support, investment in infrastructure, and collaborative efforts.

Telemedicine holds great potential to enhance remote healthcare access in India, but its successful implementation requires addressing significant challenges and barriers. Strengthening digital infrastructure, enhancing digital literacy, standardizing protocols, and developing clear regulatory frameworks are essential. Collaborative efforts and tailored approaches that respect local cultures can further facilitate telemedicine adoption. Continuous research and public awareness campaigns are necessary to ensure telemedicine's sustainable and effective use in India.

## Introduction and background

Telemedicine, a transformative aspect of healthcare delivery, has emerged as a source of innovation, breaking down geographical constraints and revolutionizing healthcare access [1]. As a fast and efficient means to exchange services between hospitals and clinics nationwide, telemedicine connects patients with medical professionals and facilitates access to education and data. Its applications range from simple requirements to complex decisions, making it a versatile and indispensable component of modern healthcare systems. Telemedicine has enhanced the ability of healthcare providers to serve a larger number of individuals without the necessity of physical presence [2]. Over recent decades, advancements in wireless broadband technology and the widespread use of cell phones and the internet have transformed telemedicine. This transformation includes patient education, medical image transfer, and real-time consultations, now a reality thanks to improved internet infrastructure and the digitalization of information, including electronic medical records (EMRs). Modern telemedicine leverages patients' and physicians' existing computing devices and affordable, self-owned equipment like smartphone cameras and wearable biosensors for clinical data collection. This user-friendly approach reduces travel expenses, saves time, cuts medical costs, and provides easier access to specialist doctors [3].

Telemedicine in India has undergone significant evolution, led by Dr. K. Ganapathy, who is recognized as the father of telemedicine in the country [4]. In 2001, he played a pivotal role in establishing the Apollo Telemedicine Networking Foundation (ATNF) and Apollo Telehealth Services, marking a milestone with the creation of the first telemedicine network connecting prestigious institutions such as the All India Institute of Medical Sciences (AIIMS), the Postgraduate Institute of Medical Education and Research (PGIMER), and the Sanjay Gandhi Postgraduate Institute of Medical Sciences (SGPGIMS) [5]. Telemedicine, focused on delivering remote clinical services through telecommunication technology, saw noteworthy developments in subsequent years: In 2003-2004, ISRO deployed satellite communication (SATCOM)-based telemedicine nodes across the country for tele-education and teleconsultation services. In 2005, the Indian Ministry of Health and Family Welfare (MoHFW) formed an Indian task force dedicated to telemedicine. In 2010, SGPGIMS was designated as the National Resource Center for Telemedicine by MoHFW, Govt. of India. In 2012, EMR/EHR standards were established by the Expert Group of MoHFW. From 2015 to 2019, telemedicine's growth, before the COVID-19 era, occurred primarily through public-private partnerships [6].

The landscape changed with the onset of the COVID-19 pandemic, leading to the release of the Telemedicine Practice Guidelines by the Government of India [7]. The eSanjeevani platform, a government-owned telemedicine initiative, emerged as a crucial tool during the pandemic, providing rapid access to healthcare services. eSanjeevaniOPD, developed within 19 days, showcased a remarkable digital transformation in health service delivery. This government initiative became a blessing, particularly for people in rural areas, ensuring access to healthcare from the comfort of their homes. The existing eSanjeevani platform that the Government of India provides under the Ayushman Bharat program operates on a hub-and-spoke model. Subcenters and Primary health centers (PHCs) serve as spokes, while district hospitals and specialist care centers function as hubs. The National Telemedicine Service in India is delivered via two variants of eSanjeevani: 'Sanjeevani AB-HWC', a doctor-to-doctor telemedicine platform, and 'eSanjeevani OPD-Stay Home OPD', a doctor-to-patient telemedicine system [7]. The success of eSanjeevani serves as a benchmark for swift capacity building and utilizing digital technology to strengthen healthcare. It aligns with the Ayushman Bharat Digital Mission, contributing to the country's integrated digital health infrastructure. In March 2023, eSanjeevani 2.0 was introduced, incorporating telediagnosis by seamlessly integrating point-of-care diagnostic devices (PoCDs), further enhancing the platform's capabilities [8]. The implementation of telemedicine services at Ayushman Bharat Health and Wellness Centers (AB-HWCs) has achieved significant milestones, enhancing healthcare accessibility across India. As of August 7, 2024, a total of 127,499 Health & Wellness Centers (HWC) operate as spokes in conjunction with over 16,211 hubs and more than 477 online OPDs. These services are supported by over 218,489 doctors, medical specialists, super-specialists, and health workers acting as telemedicine practitioners. These centers have been integrated with the eSanjeevani telemedicine platform, facilitating both doctor-to-doctor and doctor-to-patient consultations. This system allows community health officers and medical officers to consult with specialists at district hospitals, medical colleges, and institutions like AIIMS, ensuring comprehensive care while minimizing the need for physical travel.

Rationale

One of the most significant weaknesses of the healthcare system is its failure to provide care of equal quality to everyone, regardless of age, gender, ethnicity, income, geographic location, or any other demographic detail [9]. With a population exceeding 1.35 billion, India faces substantial challenges in providing equitable healthcare access. Approximately 70% of the healthcare infrastructure is concentrated in urban areas, catering to only 30% of the population. In rural areas, where 70% of the population resides, there is a glaring lack of essential physical infrastructure. The doctor-to-patient ratio in India is approximately 1:1500, significantly lower than the WHO-recommended 1:1000. It is even more skewed in rural areas at about 1:2500. Healthcare spending in India is a mere 2.1% of its GDP (2022-23), significantly below the global average of 6%. Notably, a substantial 60% of healthcare expenses in India are borne out of pocket, the highest among BRICS nations. The challenge is compounded by insufficient infrastructure and resource availability [10]. Moreover, the burden on rural healthcare is intensified by the fact that 86% of all medical visits are made by rural residents, often entailing journeys of more than 100 km. This and the predominant out-of-pocket spending underscore the need for accessible, cost-effective healthcare solutions [11]. The scenario is further complicated by the prevalent absenteeism of doctors and unpredictable closures of rural hospitals. This places an undue burden on tertiary hospitals and results in considerable hardships for patients. The consequent strain on healthcare providers and recipients underscores the critical need for innovative healthcare delivery models [1]. In this context, telemedicine emerges as a light of hope. The adoption of new and emerging information and communication technologies (ICT) promises to revolutionize healthcare delivery, making services more accessible and cost-effective, especially in unreachable populations. Hence, we wanted to conduct a scoping review to see the challenges, barriers, and facilitators in the implementation of telemedicine in India.

## Review

Methodology

This scoping review utilized the PRISMA-ScR for reporting [12].

Search Strategy

A preliminary search was conducted using PubMed only, through which key terms for the search were identified after screening titles and abstracts. The keywords and MeSH terms identified using PubMed are presented in Table 1. The literature search was conducted using PubMed and Google Scholar to identify relevant articles. It is mentioned in Table 2. In addition to the electronic database search, manual screening of reference lists and relevant systematic reviews was performed to identify any potentially overlooked studies. The search was conducted from March 4th, 2024, to March 6th, 2024, with the last search performed on March 12th, 2024, to include the most recent publications.

**Table 1:** Search terms used for preliminary database search.

Telemedicine	AND	Barriers, Facilitators	AND	India
‘telemedicine’ [MeSH Terms]		‘barrier’		‘india’[MeSH Terms]
‘digital health’[MeSH Terms]		‘challenge’		‘india’
‘telehealth’		‘obstacle’		
‘digital health care’		‘hindrance’		
‘Remote Healthcare’		‘facilitator’		
‘Virtual healthcare’		‘enabler’		
‘mHealth’		‘adoption factors’		
‘eHealth’				
‘tele-health application’				
‘telemedicine adoption’				
‘eSanjeevani OPD’				
‘virtual consultation’				
‘tele-rehabilitation’				
‘hub and spoke model’				
‘telegenetics’				

**Table 2:** Search strategy summary.

Database	Search number	Search string	Results
PubMed	#1	"telemedicine"[MeSH Terms] OR "digital health"[MeSH Terms] OR ("telemedicine"[MeSH Terms] OR "telemedicine"[All Fields] OR "telemedicine s"[All Fields]) OR (("digital health"[MeSH Terms] OR ("digital"[All Fields] AND "health"[All Fields]) OR "digital health"[All Fields]) AND "care"[All Fields]) OR ("digital health"[MeSH Terms] OR ("digital"[All Fields] AND "health"[All Fields]) OR "digital health"[All Fields]) OR "teleconsultation*"[All Fields] OR "remote health care"[All Fields] OR "virtual healthcare"[All Fields] OR "mhealth*"[All Fields] OR "ehealth*"[All Fields] OR "tele health applications"[All Fields] OR "esanjeevani OPD"[All Fields] OR "virtual consultation*"[All Fields] OR "hub spoke model"[All Fields]	137,315
	#2	"barrier*"[All Fields] OR "challenge*"[All Fields] OR "obstacle*"[All Fields] OR "hindrance*"[All Fields] OR "facilitator*"[All Fields] OR "enabler*"[All Fields] OR "adoption factor*"[All Fields]	1,470,581
	#3	"india"[MeSH Terms] OR "india*"[All Fields]	1,105,605
	#4	#1 AND #2	24,383
	#5	#1 AND #3	5,140
	#6	#1 AND #2 AND #3	1,138
		After filter (2019-2024)	925
Google Scholar	#1	with all of the words-Challenges barriers and facilitators in the Implementation of in India with the exact phrase-Telemedicine India	7
	#2	with all of the words- challenges barriers facilitators and opportunities at Primary Health care in India india telemedicine india telemedicne	15
	#3	esanjeevani barriers and challenges and facilitators in india	80
		Removing duplicates	77

Eligibility criteria

The studies were included if they were: (a) published between January 2019 and January 2024, (b) focused on telemedicine/telehealth:adoption/barriers/facilitators within the Indian healthcare setting (c) focused on telehealth/telemedicine users or providers in India, (d) investigated the role of telehealth/telemedicine in any medical specialty during the COVID-19 pandemic or after that, for telemedicine implementation and (e) were published in English. Notably, taking telehealth as a broad concept, studies were selected based on whether they used information and communication technology (ICT)-based healthcare services to diagnose, control, or manage any diseases/illnesses. However, the final analysis excluded conference abstracts, secondary data, commentaries, editorials, and brief reports containing no original data.

Title and Abstract Screening

All records identified from PubMed and Google Scholar were imported into Rayyan software. The main criteria for the title and abstract screening stage were identifying studies that addressed telemedicine or teleconsultation in India. If the study indicated such intervention in its title and abstract, it was considered relevant and included for further assessment. In case of any uncertainty regarding a study's relevance, it was still included for full-text review to avoid overlooking potentially significant studies.

Full-Text Screening

The full-text screening process involved downloading all the articles that passed the title and abstract screening stages. Each full-text article was then thoroughly assessed based on the predefined inclusion criteria to determine its eligibility for inclusion in the scoping review.

Data Extraction and Synthesis

We extracted the relevant data from the articles using Microsoft Word. Initially, we created dummy tables to facilitate the process, which included major headings such as authors' details, study settings, study location, study objectives, study design, population, sample size, and major findings pertaining to telemedicine applications, benefits, and challenges. Subsequently, significant information was extracted from each finalized article and recorded within these tables for further analysis. The extracted data is presented in Table 3 in the Appendices. The findings are summarized using the narrative synthesis approach, with implications.

Results

**Figure 1:**
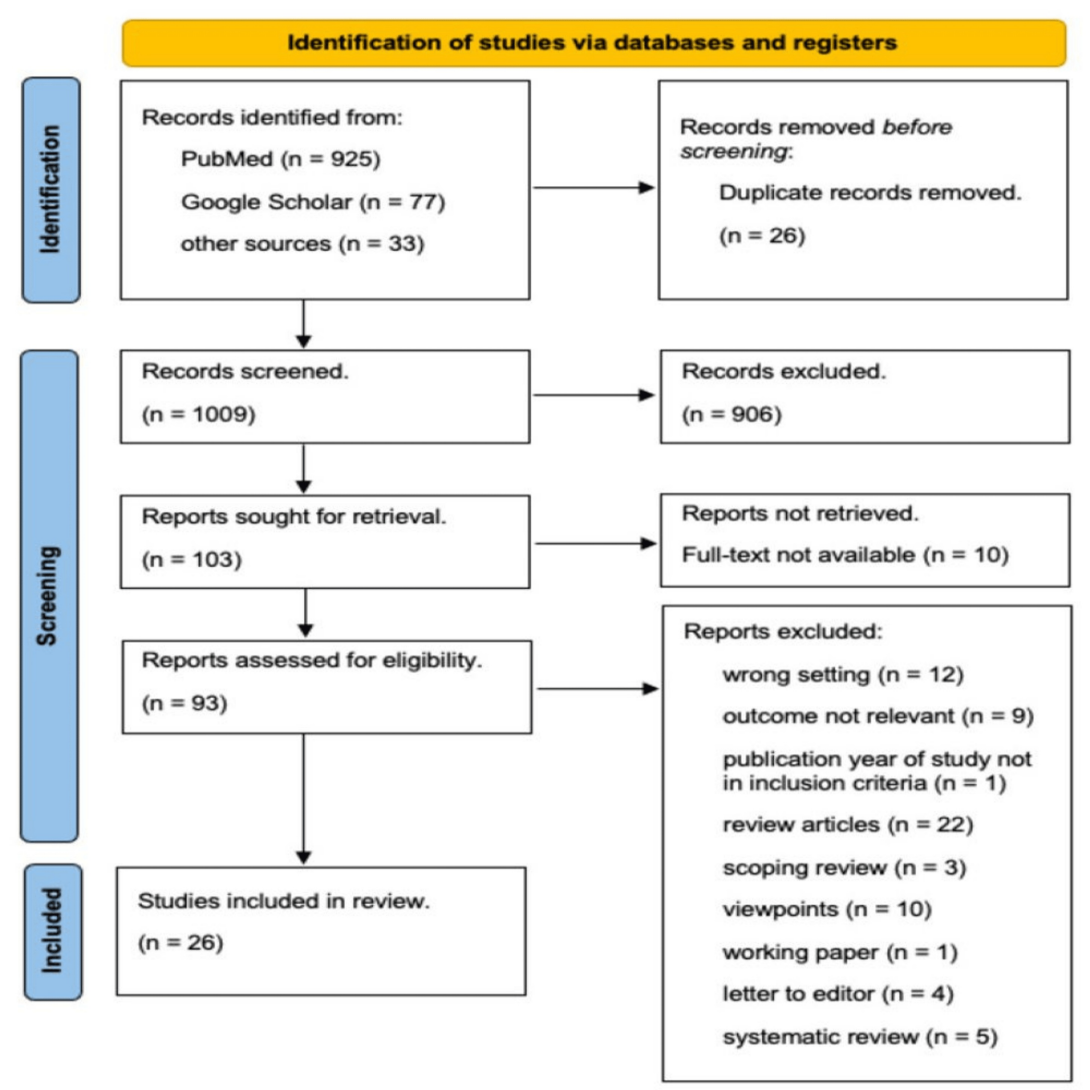
Flowchart depicting the selection process of articles included in the review.

Initial searches in PubMed, Google Scholar, and other sources identified 1035 records. Duplication resulted in 26 entries that were excluded. Of these 1009 records, 906 were excluded after screening the titles and abstracts. Out of 103 reports sought for retrieval, 10 were not retrieved because the full text was unavailable. The remaining 93 reports were assessed for eligibility, and 67 were removed because they did not meet the inclusion criteria. Thus, a total of 26 studies were included in the review [12-37]. Out of the 26 studies included, 14 are quantitative, eight are qualitative, and four are mixed methods. All the studies were conducted in different regions of India. A flowchart showing the steps involved in the entire search process is illustrated in the PRISMA-ScR (preferred reporting items for systematic reviews and meta-analyses extension for scoping reviews) 2020 diagram (Figure 1). The characteristics of the 26 studies included are given in Table 3 in the Appendices. 

Findings and discussion

This scoping review explores telemedicine's implementation challenges, barriers, and facilitators within the Indian healthcare context at different levels, focusing on its evolution and adoption from traditional settings to the current digital era.

Individual-Level Challenges, Barriers, and Facilitators

Challenges: At the individual level, the adoption of telemedicine faces significant challenges. One predominant challenge is the limited digital literacy among older adults and individuals with lower educational backgrounds, particularly in rural areas. Studies such as those by Singh et al. [14]. and Rasekaba et al. [33]. have highlighted that many patients struggle with using digital devices and navigating telehealth platforms effectively. Additionally, a lack of confidence in using technology further hinders patients from fully utilizing telemedicine services. Health literacy is another challenge, as many patients have difficulty understanding and acting upon health information provided through telemedicine consultations, as noted in the study by Gandhi et al. [28]. Mamta Manglani et al. [16] have also highlighted that technical issues and process inefficiencies led to long waiting times for teleconsultation.

Barriers: Several barriers impede the adoption of telemedicine at the individual level. Limited smartphone access and reliable internet connectivity are critical issues, especially in remote and rural areas. Studies by Raheja and Pani [15], Manish Raj et al. [26], and Mondal et al. [34] emphasize these barriers, noting that many patients in these areas lack the technology to engage in telemedicine effectively and lack experience with telemedicine. Socio-cultural factors also play a role, with many patients exhibiting a preference for face-to-face consultations and a reliance on traditional healthcare practices, as observed by Adhikari et al. [25]. and Ravindran et al. [20]. Privacy concerns, particularly in shared living environments, discourage patients from engaging fully with telehealth services.

Facilitators: Family involvement can significantly facilitate telemedicine adoption. Family members often assist with technology use and help interpret health information, as noted by Dahake et al. [18] and Singh et al. [25]. Training and educational programs aimed at enhancing both digital and health literacy among patients can also improve telemedicine adoption rates, as demonstrated in the studies by Gandhi et al. [28] and Saxena et al. [29]. Community outreach and awareness programs play a crucial role in demystifying telemedicine and encouraging its use, particularly in rural areas where digital literacy may be low, as seen in the research by Mondal et al. [34], Joseph et al. [17], and Raj and Srikanth [38].

Provider-Level Challenges, Barriers, and Facilitators

Challenges: Healthcare providers face challenges related to integrating telemedicine into their routine practices. A significant issue is the lack of training and familiarity with telehealth platforms, which can lead to suboptimal service delivery. Studies by Raheja and Pani [15] and Nair et al. [31] highlight that providers often struggle with the technology and the digital interfaces used for telemedicine. Providers also express concerns about the quality of care, particularly the inability to conduct physical examinations and the potential for misdiagnosis, as noted by Nair et al. [31] and Adhikari et al. [25].

Barriers: Insufficient infrastructure is a major barrier at the provider level, including inadequate internet bandwidth and the lack of appropriate hardware and software, as discussed by Verma et al. [20] and Ghosh et al. [22]. Medico-legal and regulatory concerns also pose significant barriers, with issues related to the legitimacy of telemedicine consultations, the legality of prescriptions, and patient data management being highlighted in studies by Nagaraja et al. [35] and Singh et al. [25]. Additionally, the absence of clear guidelines and reimbursement policies for telemedicine services complicates adoption for many healthcare providers, as noted by Agarwal and Biswas [36] and Gupta et al. [21].

Facilitators: Facilitators for providers include ongoing training and support to enhance their digital skills and familiarity with telehealth platforms, as recommended by Ghosh et al. [22] and Santhosh et al. [37]. Establishing clear guidelines and frameworks for telemedicine practice can address medico-legal concerns and streamline service delivery, as discussed by Singh et al. [27] and Gupta et al. [21]. Leveraging technology to create more user-friendly and integrated telehealth systems can also facilitate smoother adoption by healthcare providers, as shown in the research by Verma et al. [20] and Nair et al. [31]. Moreover, the study Acceptance of e-Consult for Substance Use Disorders during the COVID-19 Pandemic by Prashant Sahu et al. [23] shows high acceptability among healthcare providers, indicating trust and satisfaction with the e-consult platform. However, significant concerns about the expertise available through e-consults were noted.

System-Level Challenges, Barriers, and Facilitators

Challenges: Integrating telemedicine into existing healthcare infrastructure and ensuring its sustainability presents significant challenges at the system level. Studies by Saxena et al. [29] and Ramanadhan et al. [32] highlight the difficulties in standardizing telehealth services across different regions to ensure consistent quality of care. Additionally, maintaining patient data security and privacy across digital platforms is a critical challenge that needs to be addressed, as noted by Verma et al. [20], Rao et al. [24], and Abhishek Ghosh et al. [19].

Barriers: System-level barriers include the lack of adequate funding and resources to support telemedicine infrastructure and operations, as discussed by Verma et al. [20] and Santhosh et al. [37]. Resistance to change within healthcare institutions, where traditional practices are deeply entrenched, also poses a barrier, as observed by Raheja and Pani [15] and Saxena et al. [29]. Disparities in access to technology between urban and rural areas create inequities in telehealth service delivery, as highlighted by Mondal et al. [34] and Nair et al. [31]. The study conducted by Ravindran et al. [30] highlights the barriers to using a tele-outreach program (i.e., a telephonic call) to address psychosocial needs during the COVID-19 pandemic, such as the inability to assess non-verbal cues and various logistical issues.

Facilitators: Policy support and investment in telemedicine infrastructure, such as high-speed internet and digital health tools, can facilitate system-level adoption, as recommended by Gupta et al. [21], Ramanadhan et al. [32], and Joshi et al. [13]. Collaborative efforts between the government, healthcare institutions, and technology providers can drive the successful implementation and scaling of telehealth services, as demonstrated in the studies by Saxena et al. [29] and Gandhi et al. [28]. Creating robust frameworks for data privacy and security can build trust and ensure the safe use of telemedicine, as noted by Rao et al. [20] and Singh et al. [27].

Discussion

Individual Level

This review highlights critical areas that must be addressed to successfully implement telemedicine services in India. It starts with bolstering infrastructure, especially in rural and remote regions. Enhancing internet connectivity and digital infrastructure is crucial for the smooth delivery of telemedicine services. In partnership with private entities, government initiatives should prioritize expanding broadband and mobile network coverage to bridge the digital divide, limiting telehealth access in underserved areas. Additionally, establishing standardized protocols for telemedicine practices and providing regular training for healthcare providers is essential for ensuring consistent and effective remote care. Standardization will address variations in care practices and guarantee that patients receive high-quality services, regardless of location.

Provider Level

Enhancing digital literacy is crucial for maximizing the benefits of telemedicine. Implementing community-based programs to educate individuals, particularly in rural areas, on effectively accessing and using telehealth services is essential. Patients will be better equipped to utilize telemedicine by improving digital literacy and increasing acceptance and usage. Expanding research and monitoring alongside digital literacy underscores the importance of continuously evaluating telemedicine’s effectiveness and patient satisfaction. Ongoing research and feedback mechanisms are vital for refining telemedicine practices and addressing emerging challenges. This approach ensures that telemedicine services adapt to real-world needs and remain relevant.

System Level

Establishing clear regulatory frameworks is crucial for the success of telemedicine. These frameworks must address key legal aspects, including privacy concerns, data protection, and cross-state licensure for healthcare providers. Such legal clarity safeguards patients' rights and encourages healthcare providers to participate in telemedicine, as they can operate within well-defined guidelines and protections. In addition to regulatory clarity, integrating local cultural sensitivities and practices into telemedicine services is essential for their acceptance and effectiveness. By incorporating local traditions and languages, telemedicine can build trust and rapport between healthcare providers and patients, thereby enhancing the overall impact of these interventions. Promoting public awareness and engagement is also vital for the widespread adoption of telemedicine. Robust awareness campaigns should be launched, especially in rural areas, to highlight the benefits and availability of telemedicine. By increasing public engagement through these campaigns, telemedicine can become a well-known and trusted healthcare option, leading to its broader utilization and long-term success.

Implications

The findings of this scoping review highlight significant implications for the successful implementation of telemedicine in India. Telemedicine has the potential to bridge the healthcare access gap, particularly in underserved and rural areas, by overcoming geographical barriers and reducing the burden on traditional healthcare systems. However, the individual, provider, and system challenges must be addressed comprehensively. Enhancing digital literacy and internet accessibility, particularly in rural regions, can empower more patients to utilize telehealth services effectively. For healthcare providers, ongoing training and the development of user-friendly telehealth platforms can facilitate smoother integration into routine practice. At the system level, robust infrastructure, clear regulatory frameworks, and adequate funding are crucial for sustainable telemedicine implementation. Furthermore, considering local cultural sensitivities and fostering collaborative efforts among government, healthcare institutions, and technology providers can enhance patient trust and engagement. Continuous research and public awareness campaigns are necessary to adapt telemedicine practices to evolving needs and ensure their effective and equitable use across diverse populations in India. To address the challenges and barriers identified in this scoping review, an implementation research study can be conducted to facilitate the adoption of telemedicine in India. This study could begin with some selected primary healthcare centers, where a model can be co-developed based on context-specific strategies. Once optimized, this model can be scaled up to other areas nationwide.

Limitations

This review excludes certain study types and focuses on the literature from January 2019 to January 2024, potentially missing qualitative insights. Reliance on PubMed and Google Scholar may overlook a few relevant studies. The geographic focus might lead to overrepresentation or underrepresentation of certain areas, affecting generalizability.

## Conclusions

Telemedicine represents a transformative advancement in India's healthcare delivery system, offering significant potential to overcome geographic and infrastructure-related barriers. This scoping review identified critical challenges, barriers, and facilitators at individual, provider, and system levels. Individual-level challenges include digital and health literacy issues, while provider-level challenges involve training and quality of care concerns. System-level challenges are primarily related to infrastructure and data security. To harness telemedicine's full potential, it is imperative to strengthen digital infrastructure and connectivity, particularly in rural areas. Standardizing telemedicine protocols and providing comprehensive training for healthcare providers can address quality and service delivery concerns. Enhancing digital literacy among patients, especially in underserved regions, is also crucial. Clear regulatory frameworks and policies that address medico-legal issues and support telemedicine integration are necessary for widespread adoption. Collaborative efforts between the government, healthcare institutions, and technology providers can drive successful telemedicine implementation. Tailored approaches, considering local cultural sensitivities and practices, can improve patient engagement and trust. Sustainable models and continuous research into telemedicine's effectiveness and patient satisfaction will refine practices and address emerging challenges.

In conclusion, telemedicine can significantly enhance healthcare accessibility and quality in India. By addressing the identified challenges and leveraging the facilitators, telemedicine can be a cornerstone of a more equitable and efficient healthcare system, especially for rural and underserved populations.

## Appendices

**Table 3:** Characteristics of extracted data from Included 26 studies ANMs: Auxiliary Nurse Midwives; MPHWs: Multi-Purpose Health Workers; ASHAs: Accredited Social Health Activists; PWEs: Persons with Epilepsy

AUTHOR & YEAR	LOCATION OF STUDY	STUDY DESIGN	STUDY POPULATION	SAMPLE SIZE
(Joshi et al. 2021)	Rajasthan, India	Exploratory study	Pregnant Women and Female Children, General Population, Health Department Employees, Community Health Workers, Patients and Healthcare Consumers	Not Specified
(M. Singh et al. 2023)	Uttar Pradesh, India	Exploratory-descriptive qualitative study	Nodal officers, doctors, and patients accessing telemedicine services at 13 newly established telemedicine centres in Uttar Pradesh, India.	13 Nodal officers, 20 doctors, and 20 patients, totalling 53 participants
(Raheja and Pani, n.d.)	India	Qualitative study	Doctors practicing in various healthcare settings throughout India	40 Doctors
(Manglani et al. 2022)	Maharashtra, India.	Qualitative study	Children aged 1 to 17 years living with HIV/AIDS, their caregivers, medical officers, counsellors, and pharmacists.	48 Caregivers and 18 medical officers, counsellors, and pharmacists
(Joseph et al. 2022)	Odisha, India	Qualitative study	Parents of children with perioperative surgical care needs	26 Parents
(Dahake et al. 2023)	Nagpur, India	Descriptive qualitative study	Caregivers of children with developmental disabilities under regular follow-up	8 Caregivers of children with cerebral palsy, autism spectrum disorder, global developmental delay, and specific learning disability
(Ghosh et al. 2023)	Chandigarh, India	Qualitative study	Adult patients with substance use disorders (SUD)	15 adult patients with SUD who accessed both telemedicine and in-person care
(Verma et al. 2022)	Chandigarh, India	Observational study with an analytic survey design	Patients with hepatobiliary disorders aged 18 years or older who availed tele-hepatology services.	1,419 registrations, 1,281 completed consultations, and 210 randomly surveyed patients responded
(Gupta et al. 2023)	Jodhpur, Rajasthan	Cross-sectional study	Clinicians provide teleconsultations, and patients receive teleconsultations from the hospital's Outpatient Departments.	52 clinicians and 134 patients
(Ghosh et al. 2021)	Chandigarh, India	Descriptive study	Patients with substance use disorders	198 Patients
(Sahu et al. 2020)	India	Cross-sectional study	Healthcare providers (doctors, nurses, counsellors)	153 HCPs
(Rao et al. 2021)	Bengaluru, India	Descriptive study	Patients with genetic disorders or at risk of genetic disorders seeking tela-genetics consultation	539 Families
(Adhikari et al. 2021)	North India	Retrospective observational study	Follow-up cancer patients utilizing palliative medicine teleconsultation services	547 Patients
(M. Raj et al. 2022)	Jharkhand, India	Single-centre, cross-sectional, observational study	Patients availing teleconsultations services during the COVID-19 pandemic	758 Patients
(A. Singh et al. 2021)	Chhattisgarh, India	Cross-sectional study	Faculty members of tertiary-care teaching hospitals	115 Respondents
(Gandhi P, Kathirvel, and Chakraborty 2022)	Chandigarh, India	Cross-sectional study	ANMs, MPHWs, and ASHAs working in the study health block	80 Community health workers (ANMs, MPHWs, and ASHAs)
(Saxena et al. 2022)	Rishikesh, India	Facility-based cross-sectional study	Patients availing telemedicine consultation services during the COVID-19 pandemic	5,278 Patients
(Ravindran et al. 2020)	Bangalore, India	Descriptive study	General public affected by the COVID-19 pandemic.	Not Specified
(Nair et al. 2021)	Southern India, Tamil Nadu	Descriptive study	Persons with epilepsy (PWEs) aged 18 years and above, who have been evaluated in person within the past six months, with details available in electronic health records (EHRs), and advised regular follow-up after getting telephonic consent	Out of 336 PWE, only 141 PWE video consultation was done
(Ramanadhan et al. 2022)	Gujarat, India	Mixed-method study	Residents of Tuver village and surrounding areas.	94 Villages
(Rasekaba et al. 2022)	Karnataka, India	Mixed-method cross-sectional study	Older adults over 65 years residing in rural settings within the catchment area of JSS Hospital	150 Participants
(Mondal et al. 2023)	Kolkata, West Bengal, India	Descriptive record-based Mixed-method cross-sectional study	Users of 'Swasthya Ingit' services	Quantitative Component-data of 6775 received calls, Qualitative Component- Purposive sampling, 6 in-depth interviews (IDI) with Community Health Officers (CHO) and 5 IDIs with medical officers
(Nagaraja et al. 2024)	India	Cross-sectional internet-based survey (mix method)	Physicians in India	Quantitative Component-444 physicians And Qualitative Component-115 physicians
(Agarwal and Biswas 2020)	India	Cross-sectional, observational, web-based study	Physicians and users of mobile health applications in India	22 Mobile health applications operating in India
(Santhosh et al. 2019)	Bengaluru, India	Case Series	Three patients with tobacco addiction	3 Patients
(D. Raj and T K 2021)	Jharkhand, India.	Mixed-method study	Rural citizens, doctors in primary healthcare centres, community health workers	Not Specified
